# Functional Status, Quality of Life, and Physical Activity of Senior Club Members—A Cross-Sectional Study

**DOI:** 10.3390/ijerph19031900

**Published:** 2022-02-08

**Authors:** Ilona Stolarz, Ewelina Magdalena Baszak, Magdalena Zawadka, Piotr Majcher

**Affiliations:** 1 Department of Rehabilitation and Physiotherapy, Chair of Rehabilitation and Physiotherapy, Medical University of Lublin, 20-093 Lublin, Poland; ilona.stolarz@umlub.pl (I.S.); piotr.majcher@umlub.pl (P.M.); 2 Student Science Club of Physioprophylaxis, Department of Rehabilitation and Physiotherapy, Chair of Rehabilitation and Physiotherapy, Medical University of Lublin, 20-093 Lublin, Poland; baszak.ewelina@gmail.com; 3 Department of Sports Medicine, Chair of Clinical Physiotherapy, Medical University of Lublin, 20-093 Lublin, Poland

**Keywords:** physical activity, elderly, quality of life

## Abstract

This study aims to assess the functional status, quality of life, and physical activity (PA) of the elderly who are members of senior social clubs. The study included 63 participants (65–95 years old) who were members of a seniors club. The study utilized the following instruments: Barthel scale, Lawton’s scale of Instrumental Activities of Daily Living (IADL), WHO Quality of Life-BREF questionnaire, author’s survey questionnaire containing questions about the type of PA. Duration of membership in the senior club has a statistically significant effect on Barthel index score (F = 19.68, *p* < 0.001) and Lawton’s IADL scale results (F = 17.59, *p* < 0.001). All four domains of life quality were strongly related to the duration of attendance to senior clubs. Participants who attended the senior club for more than five years were more likely to report a longer PA duration than participants who attended the senior club for less than 5 years (Chi^2^ = 25.84, *p* < 0.001). Attendance to senior social clubs has a strong positive impact on functional status, quality of life, and PA of the elderly. Moreover, our study identified numerous social-demographic factors associated with PA, quality of life, and functional status of senior club members.

## 1. Introduction

Old age can be divided into periods as follows: early old age—between 60 and 74 years of age; late old age—between 75 and 89 years of age; very late old age—over 90 years of age [1]. Old age due to the increasing life expectancy can be a large part of life span and can be diverse in terms of physical and mental functioning. Thus, the quality of elderly life is an important social issue to improve. Functional status, on the other hand, can be defined as activities performed by an individual to realize the needs of daily living in many aspects of life [2]. The independence of the elderly can be limited due to biological processes taking place during aging, concomitant diseases, psychological factors, and physical health [3,4,5]. Poor physical and mental functioning can lead to the necessity of assistance during daily activities or institutional care and result in decreased life quality [6,7]. Quality of life is related to many factors: age, income, place of living, and education [8]. The independent factor determining poor quality of life to the greatest extent is regular exercise [9].

According to WHO recommendations, adults older than 65 years should do at least 150–300 min of moderate-intensity aerobic physical activity (PA) throughout the week, for substantial health benefits [10]. Regular aerobic activity and short-term exercise reduced the risk of functional limitations and disability in older age [11]. Sedentary behavior, otherwise, has been shown to increase the risk of diabetes, and cardiovascular disease [12]. However, regular PA has not been fully integrated into primary medical practice and is mostly limited to rehabilitation exercise during the treatment of musculoskeletal disorders in the elderly [13].

Senior social clubs can offer their members a wide variety of leisure activities. The main goal of senior clubs is the social activation of seniors and reducing loneliness among them. Through numerous forms of activities, they fulfill various functions. One of them is the integrating function. Seniors can participate in educational activities, e.g., computer skills, foreign language courses, and recreational activities, such as dance evenings, going to the cinema, trips. In addition, members have at their disposal rehabilitation classes which consist of therapeutic gymnastics, general rehabilitation training, and occupational therapy. Thanks to them, the elderly increase their functional state and learn the ergonomics of their daily activities. Previous studies showed that the users of senior clubs have higher physical function compared with the non-users and membership in the senior club leads to some improvement in health-related quality of life [14].

This study aims to assess the functional status, quality of life, and physical activity of the elderly who are members of senior social clubs. We hypothesized that longer membership can contribute to increasing independence and PA level of seniors and can be related to improvements in assessment of life quality. The findings of our study will serve a better understanding of factors associated with independence and quality of life in members of senior social clubs.

## 2. Materials and Methods

### 2.1. Participants

The study included 63 participants in age between 65 and 95 years who were members of a Seniors Club. Before data collection, each participant read and signed an informed consent document, and the study was conducted in accordance with the Declaration of Helsinki. The protocol was approved by the Ethics Committee of the Medical University of Lublin (KE/02/54/81/2021). The cross-sectional study utilized the following instruments: the Barthel scale, Lawton’s scale of Instrumental Activities of Daily Living (IADL), WHO Quality of Life-BREF (WHOQOL–BREF) questionnaire, and the author’s survey questionnaire containing questions about duration and type of PA. Group characteristics are presented in Table 1.

### 2.2. Senior Clubs

Participation in club activities is free of charge. Some of the senior clubs operated within the support centers. Recruitment to senior clubs is conducted in accordance with the principle of equal opportunities, equal treatment of men and women, and non-discrimination. Thus, participants of this study were both healthy seniors and seniors with illness or disabilities who are members of senior clubs. All clubs offer similar types of programs. There are classes of art and music, handicrafts, trips, physical exercise, games developing intellectual abilities, and memory training. The research was conducted in the period from May 2021 to July 2021 in the area of the Lubleskie Voivodeship (Poland).

### 2.3. Functional Status

The Barthel Scale assesses the independence of the respondents. It includes questions about self-sufficiency in carrying out daily activities, such as: preparing and eating meals, personal hygiene, getting dressed, changing positions, and controlling physiological functions. The maximum number of points that can be obtained is 100. Therefore, the total score ranges from 0 (completely dependent) to 100 (completely independent). The first group consisted of people with a score of 86–100 points who showed good fitness in everyday life. Respondents who could not cope with a certain part of daily activities obtained a result in the range of 21–85 points and they were placed in the second group. Full dependency is scored less than or equal to 20 points [15]. A division into groups in this study has been adopted taking into account the obtained result. The lowest score obtained in this study on the Barthel scale was 40 and the highest score was 100. Therefore, the score range was 40–100, and participants were divided into two groups: individuals who deal with activities of daily living (ADL) well (86–100 points); and those who do not deal with ADL well (40–85 points) [15].

The Lawton I-ADL scale is used to assess the independence of subjects in performing complex tasks, which include: cleaning, cooking, washing, using the telephone, DIY, shopping, and managing their money. We used the modified IADL scale based on a comprehensive geriatric assessment [16,17,18,19]. The methodology we use has been previously described by Adamek et al. [20]. Fully independent persons obtained 27 points, persons moderately dependent on the help of others obtained a result in the range of 10–26 points, and persons fully dependent obtained a result equal to or lower than 9 points [21,22].

### 2.4. Quality of Life

The WHOQOL–BREF scale Health Organization Quality of Life-Brief is used to assess the quality of life in four domains: physical and mental health, functioning in society, and social relationships. Each question has a score from 1 to 5. Each aspect can score a maximum of 20 points. The WHOQOL–BREF scale also includes two questions, assessed separately, regarding the subjective assessment of the quality of life and the satisfaction of health. Getting a higher score indicates a higher quality of life [3].

### 2.5. Physical Activity

According to WHO recommendations of PA for the elderly, participants were asked about the weekly duration of their leisure time PA (<150 min/week; about 150 min/week; >150 min/week). Moreover, seniors were asked about the most common way of spending time: sedentary (watching TV, reading, checkers, chess, crosswords); active (walking, housework, gardening, caring for family); very active (aerobic exercises, jogging, swimming, cycling, Nordic walking).

### 2.6. Statistical Analysis

Categorical data were described by absolute (N) and relative (%) frequencies, and continuous data by statistics of the mean (M), standard deviation (SD), and minimal (Min.) and maximal (Max.) values. A Chi-squared test was utilized to compare differences in categorical variables. Additionally, a Mann-Whitney U-test and one-way analysis of variance (ANOVA) were conducted to determine the differences among each demographic variable. Non-parametric Kruskal–Wallis ANOVA was used when the assumption of data normal distribution was not met. The alpha level was set at α < 0.05. All data analyses were performed using the Statistica software (ver. 13.1, TIBCO Software Inc., Palo Alto, CA, USA).

## 3. Results

### 3.1. Functional Status

Analysis of the Barthel scale results showed that females are characterized by a slightly greater Barthel index score than males (Z = −2.06, *p* = 0.04). Older participants had a lower Barthel index score than younger ones (F = 6.33, *p* < 0.01). Barthel index score was also different in terms of education (H = 15.38, *p* < 0.01), place of living (H = 10.24, *p* < 0.01), and marital status (H = 11.89, *p* < 0.01). A greater score was obtained by those who had higher education levels, lived in a big city, and are single or divorced. The lowest mean score was obtained by those seniors who graduated only primary education level, lived in a village, and are widowed. Duration of membership in the senior club has a statistically significant effect on the Barthel index score (F = 19.68, *p* < 0.001). Participants who attended the seniors club for a longer time obtained a higher score (Table 2).

Analysis of Lawton’s IADL scale results showed that females obtained better scores than males (Z = −2.54, *p*= 0.01). Older participants had a lower score on Lawton’s IADL scale than younger ones (F = 8.27, *p* < 0.001). Lawton’s IADL score was also different in terms of education (H = 9.16, *p*= 0.03), place of living (H = 6.50, *p*= 0.04) and marital status (H = 15.31, *p* < 0.01). A greater score was obtained by those who had higher education levels, lived in a small city, and were single or divorced. Duration of membership in the senior club has a statistically significant effect on Lawton’s IADL scale results (F = 17.59, *p* < 0.001). Participants who attended senior club for longer had a greater score (Table 3).

### 3.2. Quality of Life

In the physical health domain, statistically significant effects of sex (Z = −1.10, *p* = 0.04), education (H = 9.00, *p* = 0.03) and place of living (H = 10.24, *p* < 0.01) were found. The highest scores were achieved by females, college graduates, and big-city residents. Participants who attended the senior club for longer had the greatest score in the physiological health domain (F = 31.54, *p* < 0.001).

In the psychological domain, statistically, only the duration of attendance in the senior club had a statistically significant effect on the score. Participants who attend the seniors club for a longer period of time obtained the highest score in the psychological domain (F = 31.27, *p* < 0.001).

In the environment domain, statistically significant effects of place of living (H = 8.86, *p* = 0.01) and duration of senior club attendance (F = 29.18, *p* < 0.001) were found. The highest scores were achieved by big-city residents and participants who attended senior clubs longer than 5 years.

In the social relationship domain, statistically significant effects of place of living (H = 10.77, *p* < 0.01) and duration of senior club attendance (F = 19.08, *p* < 0.001) were found. The highest scores were achieved by big-city residents and participants who attended senior clubs longer than 5 years. Detailed results are shown in Table 4.

### 3.3. Physical Activity

In terms of leisure-time PA, statistically significant effect of age (Chi^2^ = 13.98, *p* < 0.01), education (Chi^2^ = 14.20, *p* = 0.03), marital status (Chi^2^ = 20.44, *p* < 0.01) and duration of senior club attendance (Chi^2^ = 25.84, *p* < 0.001) were found. Participants who were younger than 75 years, graduated from college, and were divorced most often declared that their leisure PA lasted longer than 150 min/week. Participants who attended the senior club for more than 5 years were more likely to report a longer PA duration than participants who attended the senior club for less than 5 years (Table 5).

When it comes to the way of spending free time, statistically significant effects of age (Chi^2^ = 17.73, *p* < 0.01), education (Chi^2^= 14.17, *p* = 0.03), place of living (Chi^2^ = 14.73, *p* < 0.01), and duration of senior club attendance (Chi^2^ = 28.15, *p* < 0.001) were found. Younger participants who graduated from college, lived in a big city and attended the senior club for more than 5 years reported a more active way of spending their free time (Table 6).

## 4. Discussion

### 4.1. Functional Status

This study aimed to investigate the functional status, quality of life, and physical activity of the elderly who are members of senior social clubs. We have found that attendance to the senior club is related to independence in functioning, quality in all domains of life, and can be considered a strong determinant of PA. Our results are in agreement with the stated hypothesis.

Functional status measured using Barthel and Lawton’s IADL scales was related to all investigated factors (sex, age, education, place of living, marital status). Our results are similar in some aspects to those reported previously [19,23,24]. Hachisuka et al. reported that there are no significant gender-related differences in the disability index including self-care and mobility domains in elderly persons living at home. However, there were differences between males and females and between age groups in the activity index [24]. A community-based cross-sectional study of rural elderly people conducted by Gupta et al. showed that physical disability was significantly higher among age group > 80 years, and women were more affected by physical disability than men [23]. Fidecki et al. reported that females had a slightly worse functional status than men (80.79 points vs. 80.35 points), seniors from the youngest age group showed the best fitness and marital status significantly differentiated the fitness of the respondents in terms of ADL. According to Ślusarska et al., mean value of the Barthel scale in the group of seniors provided with home care was M = 43.20 [15]. This result is significantly lower than the results of the Barthel scale of senior club members in the current study. Moreover, females reported less physical disability than males in the current study. Worth noting is the fact that in our study were no cases of great disability or total dependency among participants. Results obtained by both scales are similar and conclusive. However, we believe that it would be very interesting to compare the functioning status of senior club members, seniors who receive informal care in a home environment, and those who receive long-term institutional stationary care. This is a prospect for further research.

### 4.2. Quality of Life

All domains of life quality were strongly related to the duration of attendance to senior clubs. Through numerous forms of activities, senior social clubs fulfill various functions. Seniors can improve their skills, develop interests and meet new people. Exercises and occupational therapy are parts of senior club activities. Thanks to that, the elderly increase their functional status and learn how to cope with household duties. Only the physical health domain was significantly related to sex, education, and place of living. The psychological domain was not related to any of these factors. It was related only to the duration of attendance to senior clubs. Our findings highlight the important role of social clubs in building the psychological domain of a healthy lifestyle [25].

Interestingly, results of environment, physical health and social relationship domains were related to the place of living. The impact of the place of residence on the quality of life has been previously studied, but it is still an important topic due to the growing population of older people [26,27].

### 4.3. Physical Activity

Attendance to senior clubs is strongly related to the increasing PA of their members. Duration of PA and the way of spending free time were affected by age, education, place of living, and marital status. Our findings do not support previous research. Pettee et al. found that when compared with their single counterparts, married men and women reported a higher level of PA [28]. In our study divorced participants were more likely to do PA longer than single and married participants. Previous studies indicated that psychosocial indicators were significantly associated with older adults’ leisure-time PA [25]. We considered education and marital status as a factor affecting some of the psychosocial aspects. Qualitative studies indicate that people who had a physically demanding job, which is associated with a lower level of education, are more likely to describe retirement as a time of rest [29,30]. Higher education can be also related to greater awareness of PA health benefits.

Membership in the senior club for more than 5 years is significantly related to PA level and health behaviors. Hayashi et al. reported that participation in group exercise improved lower extremity muscle strength, but positive effects of exercise were dependent on long-term participation [31]. The users of senior centers showed higher scores in physical function than non-users [14]. In the future, it may be interesting to answer the question of whether attending seniors′ clubs may delay age-related decline in physical fitness in the elderly.

In this study, the small sample size may be considered the major limitation of the research. The recruitment of this sample was of subjects attending the senior clubs in Lublin Province from May 2021 to July 2021. Due to the COVID-19 pandemic, a questionnaire and selected research tools (scales) were made available to elderly people online. Second, our study did not examine the effects of disease on dependent variables.

## 5. Conclusions

Older people who attended classes in senior clubs for the longest time among the surveyed were much more agile, assessed their quality of life better and more often undertook physical activity than those who participated in such classes for a relatively short time. Our study identified numerous factors associated with PA, quality of life and functional status of senior club members

## Figures and Tables

**Table 1:** Baseline group characteristic.

Variable		Frequencies	
		N	%
Sex	Female	36	57.14
	Male	27	42.86
Age	65–74 years	34	53.97
	75–89 years	23	36.51
	>90 years	6	9.52
Education	Primary	12	19.05
	Vocational school	14	22.22
	High school	19	30.16
	College	18	28.57
Place of living	Village	26	41.27
	City < 50,000 inhabitants	18	28.57
	City > 50,000 inhabitants	19	30.16
Marital status	Single	6	9.52
	Married	28	44.44
	Divorced	9	14.29
	Widowed	20	31.75
Participation in senior club	About 1 year	19	30.16
	1–5 years	21	33.33
	>5 years	23	36.51
Leisure-time PA (min/week)	<150 min	18	28.57
	About 150 min	28	44.44
	>150 min	17	26.98
Way of spending time	Sedentary	13	20.63
	Active	28	44.44
	Very active	22	34.92
Barthel scale	Deal with ADL well	31	49.21
	Do not deal with ADL well	32	50.79
Lawton scale	Independency	16	25.40
	Partial dependency	47	74.60
Quality of life (WHOQOL–BREF)	Neither poor nor good	16	25.40
	Good	36	57.14
	Very good	11	17.46
Satisfaction of health (WHOQOL–BREF)	Dissatisfied	8	12.70
	Neither satisfied nor dissatisfied	24	38.10
	Satisfied	21	33.33
	Very satisfied	10	15.87

**Table 2:** Barthel scale results.

Variable		Barthel Scale			
		M	SD	Min.	Max.
Sex	Female	88.61	12.51	60	100
	Male	78.70	17.90	40	100
		Z = −2.06, ***p* = 0.04**			
Age	65–74 years	90.65	12.37	60	100
	75–89 years	83.09	14.72	50	100
	>90 years	67.50	20.68	40	95
		F = 6.33, ***p* < 0.01**			
Education	Primary	71.25	16.80	40	100
	Vocational school	79.29	18.07	50	100
	High school	87.63	11.71	60	100
	College	93.61	8.88	75	100
		H = 15.38, ***p* < 0.01**			
Place of living	Village	76.73	16.85	40	100
	City < 50,000 inhabitants	87.78	13.96	50	100
	City > 50,000 inhabitants	91.58	11.06	60	100
		H = 10.24, ***p* < 0.01**			
Marital status	Single	95	7.46	80	100
	Married	84.82	16.07	50	100
	Divorced	93.33	10.90	70	100
	Widowed	76.5	15.31	40	100
		H = 11.89, ***p* < 0.01**			
Participation in senior club	About 1 year	70.53	15.89	40	100
	1–5 years	85.71	12.87	60	100
	>5 years	94.57	7.96	70	100
		F = 19.68, ***p* < 0.001**			

M—mean; SD—standard deviation; Min.—minimal value; Max.—maximal value; Z—Mann-Whitney test; F—ANOVA; H—Kruskal–Wallis ANOVA. Bold text indicates a statistically significant comparison.

**Table 3:** Lawton’s IADL scale results.

Variable		IADL (Lawton) Scale			
		M	SD	Min.	Max.
Sex	Female	23.23	3.87	15	27
	Male	19.96	5.13	12	27
		Z = −2.54, ***p* = 0.01**			
Age	65–74 years	23.70	4.55	12	27
	75–89 years	21.65	4.26	12	27
	>90 years	15.83	2.23	13	19
		F = 8.27, ***p* < 0.001**			
Education	Primary	19.25	3.96	13	24
	Vocational school	20.43	5.12	12	27
	High school	22.89	4.28	12	27
	College	23.56	4.53	15	27
		H = 9.16, ***p* = 0.03**			
Place of living	Village	20.12	4.57	12	27
	City < 50,000 inhabitants	23.17	3.96	15	27
	City > 50,000 inhabitants	21.84	4.71	12	27
		H = 6.50, ***p* = 0.04**			
Marital status	Single	25.17	2.32	21	27
	Married	22.18	4.87	12	27
	Divorced	25	2.78	20	27
	Widowed	18.95	4.10	13	27
		H = 15.31, ***p* < 0.01**			
Participation in senior club	About 1 year	17.63	4.06	12	26
	1–5 years	22.86	3.66	17	27
	>5 years	24.39	3.70	15	27
		F = 17.59, ***p* < 0.001**			

M—mean; SD—standard deviation; Min.—minimal value; Max.—maximal value; Z—Mann-Whitney test; F—ANOVA; H—Kruskal–Wallis ANOVA. Bold text indicates a statistically significant comparison.

**Table 4:** WHOQOL–BREF scale results.

Variable		WHOQOL–BREF Scale							
		Physical Health		Psychological		Environment		Social Relationships	
		M	SD	M	SD	M	SD	M	SD
Sex	Female	15.37	2.97	15.83	2.38	15.39	2.47	15.44	2.73
	Male	13.63	3.06	15.16	2.22	14.06	2.70	14.77	2.93
		Z = −1.10 ***p* = 0.04**		Z = −1.15 *p* = 0.25		Z = −1.65 *p* = 0.10		Z = −0.85 *p* = 0.39	
Age	65–74 years	15.68	2.77	15.91	2.17	15.54	2.49	15.65	2.90
	75–89 years	14.22	3.25	15.31	2.41	14.38	2.80	14.94	2.74
	>90 years	12.86	2.47	15.45	2.54	14.50	1.82	14.45	3.09
		F = 2.73 *p* = 0.07		F = 0.460 *p* = 0.46		F = 1.40 *p* = 0.26		F = 0.64 *p* = 0.53	
Education	Primary	13.24	2.08	15.44	2.22	13.83	1.70	14.33	2.35
	Vocational school	13.35	3.69	14.62	2.62	14.18	2.76	13.90	2.85
	High school	15.07	3.05	15.61	2.34	14.82	3.00	15.86	2.63
	College	16.07	2.64	16.26	2.01	15.97	2.39	15.93	2.98
		H = 9.00 ***p* = 0.03**		H = 3.45 *p* = 0.33		H = 5.64 *p* = 0.13		H = 5.84 *p* = 0.12	
Place of living	Village	13.08	2.60	14.90	2.38	13.60	2.30	13.85	2.14
	City < 50,000 inhabitants	15.62	2.92	15.70	2.22	15.31	2.31	15.70	2.53
	City > 50,000 inhabitants	15.79	3.14	16.28	2.18	16.03	2.76	16.42	3.24
		H = 10.24 ***p* < 0.01**		H = 3.47 *p* = 0. 18		H = 8.86 ***p* = 0.01**		H = 10.77 ***p* < 0.01**	
Marital status	Single	16.48	2.06	16.56	1.76	15.42	2.82	15.56	2.75
	Married	14.61	2.96	15.15	2.34	14.76	2.73	15.14	2.49
	Divorced	17.02	2.37	16.59	1.43	16.25	1.87	17.19	1.82
	Widowed	13	2.98	14.83	2.58	14.15	2.62	14.13	3.25
		H = 9.40 *p* = 0.05		H = 4.90 *p* = 0.18		H = 6.29 *p* = 0.10		H = 7.08 *p* = 0.07	
Participation in senior club	About 1 year	11.52	2.05	13.30	1.45	12.16	1.71	13.05	2.54
	1–5 years	14.91	2.38	15.62	2.00	15.26	1.71	14.73	2.09
	>5 years	16.92	2.16	17.33	1.43	16.61	2.21	17.28	2.11
		F = 31.54 ***p* < 0.001**		F = 31.27 ***p* < 0.001**		F = 29.18 ***p* < 0.001**		F = 19.08 ***p* < 0.001**	

M—mean; SD—standard deviation; Z—Mann–Whitney test; F—ANOVA; H—Kruskal–Wallis ANOVA. Bold text indicates a statistically significant comparison.

**Table 5:** Leisure-time physical activity.

Variable		Leisure-Time PA (per Week)			
			<150 min	About 150 min	>150 min
Sex	Female	N	9	16	11
		%	25.00%	44.44%	30.56%
	Male	N	9	12	6
		%	33.33%	44.44%	22.22%
		Chi^2^ = 0.77, *p* = 0.68			
Age	65–74 years	N	3	10	10
		%	13.04%	43.48%	43.48%
	75–89 years	N	10	17	7
		%	29.41%	50.00%	20.59%
	>90 years	N	5	1	0
		%	83.33%	16.67%	0.00%
		Chi^2^ = 13.98, ***p* < 0.01**			
Education	Primary	N	7	4	1
		%	58.33%	33.33%	8.33%
	Vocational school	N	3	10	1
		%	21.43%	71.43%	7.14%
	High school	N	5	7	7
		%	26.32%	36.84%	36.84%
	College	N	3	7	8
		%	16.67%	38.89%	44.44%
		Chi^2^ = 14.20, ***p* = 0.03**			
Place of living	Village	N	11	13	2
		%	43.31%	50.00%	7.69%
	City < 50,000 inhabitants	N	3	7	8
		%	16.67%	38.89%	44.44%
	City > 50,000 inhabitants	N	4	8	7
		%	21.05%	42.11%	36.84%
		Chi^2^ = 9.62, *p* = 0.05			
Marital status	Single	N	2	2	2
		%	33.33%	33.33%	33.33%
	Married	N	7	13	8
		%	25.00%	46.43%	28.57%
	Divorced	N	0	2	7
		%	0.00%	22.22%	77.78%
	Widowed	N	9	11	0
		%	45.00%	55.00%	0.00%
		Chi^2^ = 20.44, ***p* < 0.01**			
Participation in senior club	About 1 year	N	12	7	0
		%	63.16%	36.84%	0.00%
	1–5 years	N	2	14	5
		%	9.52%	66.67%	23.81%
	>5 years	N	4	7	12
		%	17.39%	30.43%	52.17%
		Chi^2^ = 25.84, ***p* < 0.001**			

Chi^2^—Chi-squared test. Bold text indicates a statistically significant comparison.

**Table 6:** Ways of spending free time.

Variable		Way of Spending Time			
			Very Active	Active	Sedentary
Sex	Female	N	14	15	7
		%	38.89%	41.67%	19.44%
	Male	N	8	13	6
		%	29.63%	48.15%	22.22%
		Chi^2^ = 0.58, *p* = 0.75			
Age	65–74 years	N	14	7	2
		%	60.87%	30.43%	8.70%
	75–89 years	N	8	19	7
		%	23.53%	55.88%	20.59%
	>90 years	N	0	2	4
		%	0.00%	33.33%	66.67%
		Chi^2^ = 17.73, ***p* < 0.01**			
Education	Primary	N	0	7	5
		%	0.00%	58.335	41.67%
	Vocational school	N	4	6	4
		%	28.57%	42.86%	28.57%
	High school	N	7	9	3
		%	36.84%	47.37%	15.79%
	Collage	N	11	6	1
		%	61.11%	33.33%	5.56%
		Chi^2^ = 14.17, ***p* = 0.03**			
Place of living	Village	N	3	14	9
		%	11.54%	53.85%	34.62%
	City < 50,000 inhabitants.	N	7	9	2
		%	38.89	50.00%	11.11%
	City > 50,000 inhabitants	N	12	5	2
		%	63.16%	26.32%	10.53%
		Chi^2^ = 14.73, ***p* < 0.01**			
Marital status	Single	N	3	3	0
		%	50.00%	50.00%	0.00%
	Married	N	10	13	5
		%	35.71%	43.43%	17.86%
	Divorced	N	6	3	0
		%	66.67%	33.33%	0.00%
	Widowed	N	3	9	8
		%	15.00%	45.00%	40.00%
		Chi^2^ = 12.42, *p* = 0.05			
Participation in senior club	About 1 year	N	0	9	10
		%	0.00%	47.37%	52.63%
	1–5 years	N	7	12	2
		%	3.33%	57.14%	9.52%
	>5 years	N	15	7	1
		%	65.22%	30.43%	4.35%
		Chi^2^ = 28.15, ***p* < 0.001**			

Chi^2^—Chi-squared test. Bold text indicates a statistically significant comparison.

## Data Availability

Not applicable.
